# Role of synthetic retinoic acid derivatives in Chronic Lymphocytic Leukemia and Multiple Myeloma

**DOI:** 10.1186/1753-6561-7-S1-O3

**Published:** 2013-01-30

**Authors:** Ayman T Saeed, P Murphy, S Napoletano

**Affiliations:** 1 Royal College of Surgeons in Ireland, Dublin, Ireland; 2 Beaumont Hospital, Dublin, Ireland

## Background

B-cell Chronic Lymphocytic Leukemia (CLL) is the most common leukemia diagnosed in adults in the western world. Multiple Myeloma (MM), a plasma cell neoplasm based in bone-marrow, comprises about 1% of all malignant tumours. These are two incurable disorders with treatments that focus on controlling the disease and symptoms rather than eradicating it. Synthetic analogues of Retinoic Acid (RA) are currently used extensively for the treatment of skin disorders such as acne vulgaris and psoriasis, as well certain forms of cancer namely Acute Promyelocytic Leukemia. Preclinical studies have also garnered support for their chemopreventive potential in many more cancers, especially haematological malignancies.

The purpose of this study was to illustrate the roles of Fenretinide (4HPR) and Acitretin in CLL and MM and establish whether the two retinoic acid derivatives may prove to be prospective treatments for these malignancies. Fenretinide (4HPR) is a drug studied in a variety of cancers and has shown to induce apoptosis through increased levels of Reactive Oxygen Species (ROS) and activation of caspase-8, 9 and 3. Acitretin is currently used to treat psoriasis but little is known about its effect on cancer. Therefore, its mechanism of action is unknown.

## Results

For the purpose of this study, 5 MM cell lines (RPMI-8226, NCI-H929, MM1.s, KMS-BM-12, U-266) and primary cells isolated from whole blood of CLL patients were used. 4HPR significantly decreased cell viability in 4 out of 5 MM cell lines tested at 5, 10, 20μM, whereas Acitretin only had an effect at concentrations higher than 50uM in 2 MM cell lines. CLL cells were highly sensitive to 4HPR at all concentrations used, but were only sensitive to Acitretin up until 10μM. 4HPR and Acitretin also significantly decreased NCI-H929 and CLL cells migration capabilities. Additionally, western blot analysis on RPMI-8226 cells showed that 4HPR and Acitretin negatively affected the expression of the cell cycle markers cyclin D2 and phospho-Rb, the anti-apoptosis marker Mcl-1, and increased cleavage and therefore activation of the pro-apoptotic marker PARP. This suggests that the two inhibitors could affect both cell proliferation and apoptosis.

## Conclusions

Having conveyed promising cytotoxic and chemoattractant properties with both CLL and MM cells, 4HPR and Acitretin have proven to be promising therapeutic candidates for MM and CLL treatment.

